# Phytochemical Analysis and Free Radical Scavenging Activity of Medicinal Plants *Gnidia glauca* and *Dioscorea bulbifera*

**DOI:** 10.1371/journal.pone.0082529

**Published:** 2013-12-18

**Authors:** Sougata Ghosh, Abhishek Derle, Mehul Ahire, Piyush More, Soham Jagtap, Suvarna D. Phadatare, Ajay B. Patil, Amit M. Jabgunde, Geeta K. Sharma, Vaishali S. Shinde, Karishma Pardesi, Dilip D. Dhavale, Balu A. Chopade

**Affiliations:** 1 Institute of Bioinformatics and Biotechnology, University of Pune, Pune, India; 2 National Centre For Free Radical Research, Department of Chemistry, University of Pune, Pune, India; 3 Garware Research Centre, Department of Chemistry, University of Pune, Pune, India; 4 Department of Microbiology, University of Pune, Pune, India; University of Sassari, Italy

## Abstract

*Gnidia glauca* and *Dioscorea bulbifera* are traditional medicinal plants that can be considered as sources of natural antioxidants. Herein we report the phytochemical analysis and free radical scavenging activity of their sequential extracts. Phenolic and flavonoid content were determined. Scavenging activity was checked against pulse radiolysis generated ABTS^•+^ and OH radical, in addition to DPPH, superoxide and hydroxyl radicals by biochemical methods followed by principal component analysis. *G. glauca* leaf extracts were rich in phenolic and flavonoid content. Ethyl acetate extract of *D. bulbifera* bulbs and methanol extract of *G. glauca* stem exhibited excellent scavenging of pulse radiolysis generated ABTS^•+^ radical with a second order rate constant of 2.33×10^6^ and 1.72×10^6^, respectively. Similarly, methanol extract of *G. glauca* flower and ethyl acetate extract of *D. bulbifera* bulb with second order rate constants of 4.48×10^6^ and 4.46×10^6^ were found to be potent scavengers of pulse radiolysis generated OH radical. *G. glauca* leaf and stem showed excellent reducing activity and free radical scavenging activity. HPTLC fingerprinting, carried out in mobile phase, chloroform: toluene: ethanol (4: 4: 1, v/v) showed presence of florescent compound at 366 nm as well as UV active compound at 254 nm. GC-TOF-MS analysis revealed the predominance of diphenyl sulfone as major compound in *G. glauca*. Significant levels of n-hexadecanoic acid and octadecanoic acid were also present. Diosgenin (C_27_H_42_O_3_) and diosgenin (3á,25R) acetate were present as major phytoconstituents in the extracts of *D. bulbifera. G. glauca* and *D. bulbifera* contain significant amounts of phytochemicals with antioxidative properties that can be exploited as a potential source for herbal remedy for oxidative stress induced diseases. These results rationalize further investigation in the potential discovery of new natural bioactive principles from these two important medicinal plants.

## Introduction

Oxidative stress, a key player in several diseases such as cancer, diabetes mellitus, atherosclerosis, cardiovascular diseases, ageing and inflammatory diseases, results from an imbalance between formation and neutralization of prooxidants [1]. Oxidative stress is initiated by free radicals, which seek stability through electron pairing with biological macromolecules such as proteins, lipids and DNA in healthy human cells and cause protein and DNA damage along with lipid peroxidation [2]–[4]. Enzymes, particularly superoxide dismutase (SOD) and catalase as well as compounds like tocopherol, ascorbic acid and glutathione play a key role in protecting human cells from free radical mediated damage [5]. In conditions, where free radical production rate may exceed, the capacity of antioxidant defense mechanisms results in substantial tissues injury. Antioxidant principles from medicinally important plants possess enormous potential in correcting imbalance mediated oxidative stress and various degenerative diseases [6]. Recently, much attention has been directed towards development of ethnomedicines with strong antioxidant properties but low cytotoxicity. Additionally, it has been determined that antioxidant effect of plant products is mainly due to radical-scavenging activity of phenolic compounds such as flavonoids, polyphenols, tannins, and phenolic terpenes [7]–[9]. Indian system of Ayurvedic medicine mentions many herbal medicines for treating various diseases like diabetic mellitus, rheumatoid arthritis and cardiovascular diseases [10]. We selected two plants, *Gnidia glauca* and *Dioscorea bulbifera,* to explore their antioxidant activity.

*G. glauca* is an important medicinal plant which is used in traditional medicine for wound healing and viral infections [11]–[14]. Although this plant has numerous applications on herbal remedy, till date there are no scientific evidences behind the mechanisms.

*D. bulbifera* commonly known as yam or air potato is also a medicinal plant which is extensively used in treatment of gastric cancer and carcinoma of rectum, goiter and sore throat. Various extracts of bulbs have been reported to be antihyperlipidemic, antitumor, antioxidant, anorexiant, analgesic, anti-inflammatory, plasmid curing and antihyperglycemic [15], [16].

Recently, we have reported for the first time on detailed mechanism of antidiabetic potential of both *G. glauca* and *D. bulbifera* as well as their applications in nanobiotechnology [16]–[19]. Phytochemical constituents are significant for overall biological activity. In view of this background, there is a growing interest to investigate the unexplored potential of these endemic medicinal plants found in Western Ghats of Maharashtra, India.

Objective behind the present study was to carry out phytochemical analysis and evaluate free radical scavenging activity of both *G. glauca* and *D. bulbifera* extracts. Extracts were examined for total phenolic and flavonoid content. Potential for scavenging of different reactive oxygen species (ROS) including hydroxyl, superoxide and nitric oxide was also evaluated. Herein we report for the first time, HPTLC fingerprinting and complete phytochemical profiling employing GC-TOF-MS for different extracts of *G. glauca* and *D. bulbifera.*

## Materials and Methods

### Chemicals and Reagents

Folin–Ciocalteu reagent and quercetin were obtained from Qualigens, Mumbai, India. Gallic acid, L-ascorbic acid, potassium thiocyanate, ethylene diamine tetra acetic acid (EDTA) 2-2′-azinobis 3-ethylbenzothioline-6-sulfonic acid (ABTS), 2,2- diphenyl-1-picrylhydrazyl (DPPH), 2, 4, 6-[Tri(2-pyridyl)-s-triazine] (TPTZ), phenazine methosulfate (PMS), nitroblue tetrazolium (NBT), riboflavin, 2-deoxyribose, thiobarbituric acid (TBA), sodium nitroprusside, sulphanilic acid, N-(1-Naphthyl) ethylenediamine dihydrochloride, potassium hexacyanoferrate (K_3_Fe(CN)_6_), trichloroacetic acid (TCA), ferric chloride were procured from HiMedia Laboratories, Mumbai, India.

### Plant material and preparation of extracts

Fresh mature leaves, stems, and flowers of *G. glauca* (voucher specimen number 327) and bulbs of *D.bulbifera* (voucher specimen number 860) were collected in month of January from Western Ghats of Nashik and Sinhagad hills region of Maharashtra, India, which were identified and authenticated by botanist from National Research Institute of Basic Ayurvedic Sciences, Central Council for Research in Ayurveda and Siddha, Department of Ayush, Ministry of Health and Family Welfare, Government of India, New Delhi, Nehru Garden, Kothrud, Pune, India. Extracts of leaves, stems and flowers of *G. glauca* and bulbs of *D. bulbifera* were prepared as per the process reported earlier [16]. In short, plant materials were shade dried at room temperature upto one week. Dried plant materials were reduced to powder by using an electric blender, 100 g of which was subjected to a cold extraction with 70% (v/v) ethanol in distilled water as well as sequentially extracted with petroleum ether, ethyl acetate and methanol. Petroleum ether, ethyl acetate and methanol extracts were evaporated to dryness under reduced pressure at 40°C in rotary evaporator while hydroalcoholic extract was subjected to lyophilization and were stored in air-tight containers in refrigerator at 4°C. Extracts were further reconstituted to get a final concentration of 1mg/mL which was used in all biochemical assays. Ascorbic acid (1 mg/mL) was used a reference standard while methanol was used a control in all the experiments.

### Ethics statement

Specific permissions were not required for the described field sampling studies or for the collection of plants materials. For any locations/activities, no specific permissions were required. All locations where the plants were collected were not privately-owned or protected in any way and the field studies did not involve endangered or protected species.

### Polyphenolic content

125 µL of sample was mixed with 500 µL of distilled water and 125 µL of 25% Folin–Ciocalteu reagent which was allowed to react for 5 min followed by addition of 1.25 mL of 7% Na_2_CO_3_. Thereafter, it was thoroughly mixed and placed in darkness for 1.5 h and absorbance was measured at 760 nm using UV/Visible spectrophotometer. Total phenolic content was quantified from gallic acid standard curve [18].

### Flavonoid content

Total flavonoid content was quantified according to Luximon - Ramma (2002) with a minor modifications [18], [20]. In brief, 100 µL of sample and 100 µL of 2% aluminum chloride was mixed together followed by incubation for 10 min at room temperature. Absorbance of reaction mixture was measured at 368 nm with UV/Visible spectrophotometer. Flavonoid content was evaluated from calibration curve of quercetin, a standard flavonoid.

### Pulse radiolysis generated hydroxyl radical scavenging assay

Hydroxyl radicals (**^•^**OH) were formed by radiolysis of water in linear accelerator (LINAC) electron pulse radiolysis system at ‘National Center for Free Radical Research (NCFRR), University of Pune, Pune, India. Irradiation of water with 7 MeV electron pulse (100 ns pulse width) and dose rate 17 Gy/pulse generated hydroxyl radicals, hydrated electrons and hydrogen atoms. In order to measure only reactions of **^•^**OH, all solutions were pre-saturated with nitrous oxide (N_2_0) for removal of dissolved oxygen. Generated hydroxyl radicals were made to react with extracts. First order rate constants for radical formation were measured and found to vary with plant extracts. Slope of linear plots gave second order rate constants. Ability to scavenge hydroxyl radicals was measured by comparing it with standard potassium thiocyanate (KSCN) using competition kinetics [21]. In this method, **^•^**OH is made to react with 1 mM KSCN in absence and in presence of plant extracts. **^•^**OH reacts completely with SCN^−^ to produce (SCN)_2_^−^ which absorbs at 480 nm. In presence of extracts decrease in absorbance was measured. Difference between rate constant of (SCN)_2_ **^•^** ^−^ was calculated.

Alternatively, 100 µL of extract was added to 400 µL of phosphate buffer (50 mM, pH 7.4), 100 µL of EDTA (1.04 mM), 100 µL of FeCl_3_ (1.0 mM) and 100 µL of 2-deoxyribose (60 mM). Mixtures were kept in water bath at 37°C and reaction was initiated by addition of 100 µL of ascorbic acid (2 mM) and 100 µL of H_2_O_2_ (10 mM). After 1 hr, 1 mL of cold thiobarbituric acid (10 g/L) was added into reaction mixture followed by 1 mL of HCl (25%) and kept in boiling water bath at 100°C for 15 min. Absorbance was measured at 532 nm [22]. Hydroxyl radical scavenging capacity was evaluated by following formula:
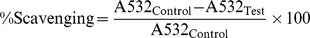



### Pulse radiolysis generated ABTS^• +^ radical scavenging assay

Scavenging of ABTS ^+^ radical by plant extracts was determined using pulse radiolysis [21]. Reaction mixture (4 mL) contained 0.05 M sodium azide (NaN_3_), 2 mM 2-2′-azinobis 3-ethylbenzothioline-6-sulfonic acid (ABTS) and distilled water. After purging with N_2_O for 5 min, samples were exposed to an electron beam of pulse width 100 ns at a dose rate of 17.6 Gy/pulse. ABTS^•+^ radical was produced by reaction of radiolytically generated azide radicals with ABTS^2^. Scavenging of radical was estimated by recording the absorbance at 600 nm.

### DPPH radical scavenging assay

20 µL of each extract were mixed with 80 µL of methanolic solution of 2,2- diphenyl-1-picrylhydrazyl (DPPH, 100 µM) in 96 well plate followed by incubation in darkness at room temperature for 30 min [23]. Change in absorbance was measured at 517 nm in a 96-well plate reader (SpectraMax M5, Molecular Devices Corporation, Sunnyvale, CA). Radical scavenging activity was found out by following formula:
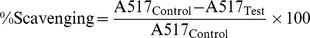



### Ferric reducing antioxidant property

FRAP solution was freshly prepared by mixing 25 ml of 300 mM acetate buffer, 2.5 mL of 10 mM TPTZ solution and 2.5 mL of 20 mM FeCl_3._.6H_2_O solution. 30 µl of extract was allowed to react with 900 µL of FRAP solution followed by an incubation for 15 min in darkness [24]. Absorbance measured at 595 nm was used to quantify the activity by extrapolating from standard calibration curve. Percentage scavenging was expressed in terms of gallic acid equivalent antioxidant capacity (GAEAC). Concentration dependent Fe^3+^ reducing power of extracts was determined by method of Tan *et al*, (2011) [25]. 750 µL of extract was mixed with 750 µL of phosphate buffer (0.2 M, pH 6.6) and 750 µL of potassium hexacyanoferrate (K_3_Fe(CN)_6_) (1%, w/v), followed by incubation at 50°C in a water bath for 20 min. Reaction was stopped by adding 750 µL of trichloroacetic acid (TCA) solution (10%) and then centrifuged at 3000 rpm for 10 min. 1.5 mL of supernatant was mixed with 1.5 mL of distilled water and 100 µL of ferric chloride (FeCl_3_) solution (0.1%, w/v) for 10 min. Absorbance at 700 nm was measured as reducing power. Higher absorbance of reaction mixture indicated greater reducing power.

### Superoxide anion scavenging assay

Superoxide anions were generated in a non-enzymatic phenazine methosulfate-nicotinamide adenine dinucleotide (PMS-NADH) system through reaction of PMS, NADH, and oxygen indicated by reduction of nitroblue tetrazolium (NBT) [26]. 300 µL of extract was added in 3 mL of Tris-HCl buffer (100 mM, pH 7.4) containing 750 µL of NBT (300 µM) solution and 750 µL of NADH (936 µM) solution. Reaction was initiated by adding 750 µL of PMS (120 µM) to the mixture. After 5 min of incubation at room temperature, absorbance at 560 nm was measured in spectrophotometer. Superoxide anion scavenging activity was calculated according to following equation:
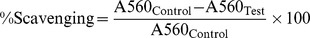



Alternatively, 100 µL of extract was added to 100 µL riboflavin solution (20 µg), 200 µL EDTA solution (12 mM), 200 µL ethanol and 100 µL NBT solution (0.1 mg). Reaction mixture was diluted up to 3 mL with phosphate buffer (50 mM) followed by illumination for 5 min. Absorbance of solution was measured at 540 nm [26]

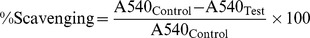



### Nitric oxide scavenging activity assay

2 mL of 10 mM sodium nitroprusside in 500 µL phosphate buffer saline (pH 7.4) was mixed with 500 µL of extract followed by incubation at 25°C for 150 min. 500 µL of above mixture was taken out and added into 1 mL sulphanilic acid reagent (33% in 20% glacial acetic acid) and incubated at room temperature for 5 min. Finally, 1 mL naphthylethylenediamine dihydrochloride (0.1% w/v) was mixed and incubated at room temperature for 30 min before measuring the absorbance at 540 nm [27]. Percentage nitric oxide scavenging activity was calculated using following equation:
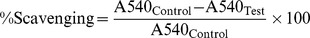



### HPTLC fingerprint profiles for various extracts

TLC plate consists of 10*×*10 cm, precoated with silica gel 60 F254 TLC plates (E.Merck, Germany) (0.2 mm thickness) with aluminum sheet support. Spotting device was a CAMAG Linomat V Semi-automatic Sample Spotter (Camag Muttenz, Switzerland); syringe, 100 µL (from Hamilton by CAMAG); developing chamber was a CAMAG glass twin trough chamber (10*×*10 cm); densitometer consisted of a CAMAG TLC scanner 4 linked to WINCATS software 1.4.6. Mobile phase was chloroform: toluene: ethanol (4: 4: 1, v/v). Saturation time for mobile phase was 20 min. 10 µL of all extracts were applied on TLC plate and developed in solvent system to a distance of 8 cm. Plates were dried at room temperature in air followed by scanning at 254 nm and 366 nm. After spraying with anisaldehyde sulfuric acid reagent plates were heated at 110°C for 5 min and scanned at 600 nm. *Rf* values and color of resolved bands were noted.

### GC-TOF-MS analyses

In this study, measurements were made with a LECO Pegasus 4D GCxGC-TOFMS system that consists of an Agilent 6890 gas chromatograph equipped with a LECO dual-jet thermal modulator between primary and secondary columns and a LECO Pegasus IV Time-of-Flight Mass Spectrometer (TOFMS) as a detector. Primary analytical column was a HP-5MS capillary column (5% phenyl polysilphenylene-siloxane; 30 m×0.32 mm, 0.25 µm). Secondary column was a 1.00 m×0.10 mm ID×0.10 µm of RXI-17ms which was housed in GC oven. Gerstel PTV using solvent vent mode was used for injecting samples. Modulator temperature offset for this study was +20°C. Helium was used as carrier gas at a ramped pressure mode. Transfer line was kept at 240°C. Primary oven program was initially set at 100°C for 0.5 min, followed by an increase up to 215°C at an increment of 20°C/min, held for 0.5 min, thereafter to 270°C at 25°C/min and held for 10 min. For secondary oven program, rate and duration were identical to primary oven. However, target temperature was set at 30°C above primary oven. MS-parameters for Pegasus GC-TOFMS had electron impact ionization at 70 eV, and ion source temperature was 250°C. Detector voltage was set at 1700 V, and data acquisition was carried out within mass range of *m*/*z* 50–500 at an acquisition rate of 10 spectra. Software ChromaTOF 3.34 was used for data processing and automatic assignment of peaks and integrations. Identification of components was based on comparison of their mass spectra with those of NIST library spectra (v. 2.0) [28].

### Statistical analysis

Statistical analysis was performed using one way analysis of variance (ANOVA) and two tailed t-test (P<0.05). Results are expressed as means ± SEM (n = 3). Antioxidant activity and effects of extracts in different solvents were subjected to principle component analysis (PCA).

## Results

### Phenolic content

Phenolic contents were found to be significantly high (*P*<0.05) in methanolic extracts (Table 1). Among petroleum ether extracts, phenolic content of *G. glauca* stem was found to be maximum followed by flower. Leaf of *G. glauca* showed high amount of polyphenols. Among various extracts of *D. bulbifera*, phenolic content of methanol extract was found to be maximum while both ethyl acetate and 70% (v/v) ethanol extracts were in a range between 80 to 100 µg/mL.

**Table 1:** Total phenolic content of plant extracts.

	Total phenolic content (µg/mL)			
Plant extract	Petroleum ether	Ethyl acetate	Methanol	Ethanol (70% v/v)
*G.glauca*				
Flower	54.55±0.48	169.33±0.84	174.33±0.69*	143.00±0.69
Leaf	51.56±1.57	174.00±0.84	213.44±5.03*	151.11±1.93
Stem	64.00±0.77	139.78±1.5	164.56±0.97*	139.89±0.95
*D.bulbifera*				
Bulb	49.22±0.80	98.00±1.17	145.44±3.29*	85.89±1.16

The data is indicated as the mean ± SEM; [*n = *3]. Data with asterisk (*) shows significant difference (*P*<0.05), two-tailed student t-test.

### Flavonoid content

Total flavonoid present in petroleum extracts were found in a range of 3 to 10 µg/mL (Table 2). *G. glauca* flower showed highest flavonoid content among petroleum ether extracts. In case of ethyl acetate and 70% (v/v) ethanolic extracts, leaf of *G. glauca* even showed a significantly high flavonoid content (*P*<0.05) than stem and flower. Flavonoid content in *D. bulbifera* bulbs was in a range of 4 to 30 µg/mL, highest being ethyl acetate.

**Table 2:** Total flavonoid content of plant extracts.

	Total flavonoid content(µg/mL)			
Plant extract	Petroleum ether	Ethyl acetate	Methanol	Ethanol (70% v/v)
*G.glauca*				
Flower	7.48±0.38	35.62±0.40	53.05±0.42	21.69±0.35
Leaf	4.36±0.04	92.76±0.36*	62.98±0.48	115.38±0.40*
Stem	3.71±0.04	10.86±0.72	21.90±0.81	10.29±0.65
*D.bulbifera*				
Bulb	4.95±0.10	27.86±0.18	12.76±0.48	12.10±0.05

The data is indicated as the mean ± SEM; [*n = *3]. Data with asterisk (*) shows significant difference (*P*<0.05), two-tailed student t-test.

### Pulse radiolysis generated hydroxyl radical scavenging activity

**Figure 1:**
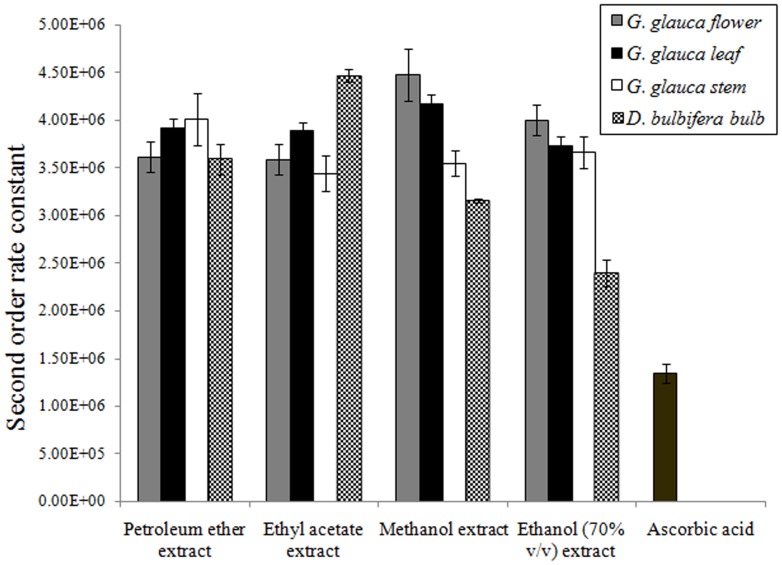
Pulse radiolysis generated OH radical scavenging by plant extracts.

Pulse radiolysis generated hydroxyl radical scavenging activity of compounds was checked. Second order rate constant for reac­tion of any antioxidant with free radicals indicates its reactivity towards free radical (Figure 1). All of the tested extracts were found to have excellent activity as compared to ascorbic acid (1.34×10^6^) which was used as a standard. Petroleum ether extract of *G. glauca* stem (4.01×10^6^), was most superior as compared to *G. glauca* leaf (3.92×10^6^) while *G. glauca* flower (3.61×10^6^) and *D. bulbifera* bulb (3.59×10^6^) showed moderate activity. Ethyl acetate extract of *D. bulbifera* bulb (4.46×10^6^) showed maximum activity. Methanolic extracts of *G. glauca* flower (4.48×10^6^), leaf (4.18×10^6^) and stem (3.55×10^6^) showed excellent activity. Similarly, ethanolic extracts of *G. glauca* flower showed maximum activity (4×10^6^), followed by its leaf (3.73×10^6^) and stem (3.66×10^6^) where as *D. bulbifera* (2.4×10^6^) exhibited comparatively lower activity. In the alternate method as well, plant extracts showed excellent hydroxyl radical scavenging activity (Table 3). *G. glauca* leaf exhibited potent antioxidant activity with all three extracts, highest being methanol. Among the extracts of *D. bulbifera* bulb, methanolic extract showed excellent activity (*P*<0.05) followed by ethyl acetate and 70% (v/v) ethanolic extracts.

**Table 3:** Hydroxyl radical scavenging activity of plant extracts.

Plant extract	% Hydroxyl radical scavenging activity			
AA = 77.52±0.39	Petroleum ether	Ethyl acetate	Methanol	Ethanol (70% v/v)
*G.glauca*				
Flower	46.50±1.43	77.01±0.65	78.16±0.96*	74.69±0.72
Leaf	44.77±1.03	77.97±1.20	81.25±0.34*	77.46±0.22
Stem	41.30±0.72	70.58±1.19	74.69±0.61*	72.83±0.51
*D.bulbifera*				
Bulb	44.51±0.49	66.67±0.73	76.11±1.26*	64.23±1.25

AA = Ascorbic acid; the data is indicated as the mean ± SEM; [*n = *3]. Data with asterisk (*) shows significant difference (*P*<0.05), two-tailed student t-test.

### Pulse radiolysis generated ABTS^• +^ radical scavenging activity

**Figure 2:**
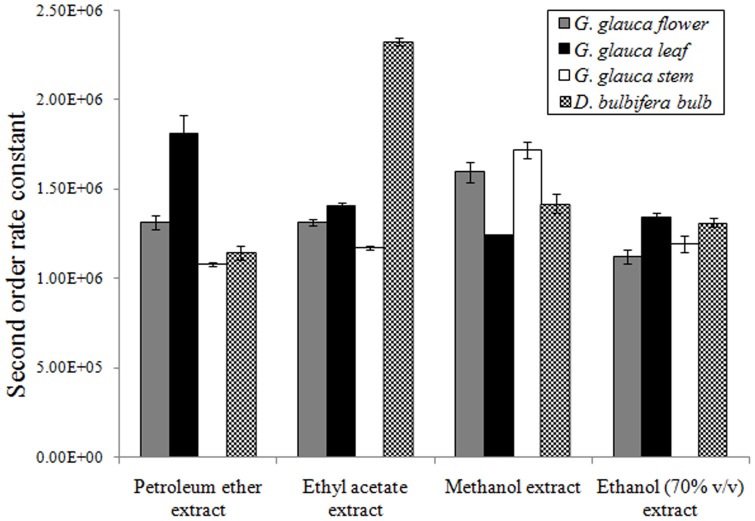
Pulse radiolysis generated ABTS^• +^ radical scavenging by plant extracts.

**Figure 3:**
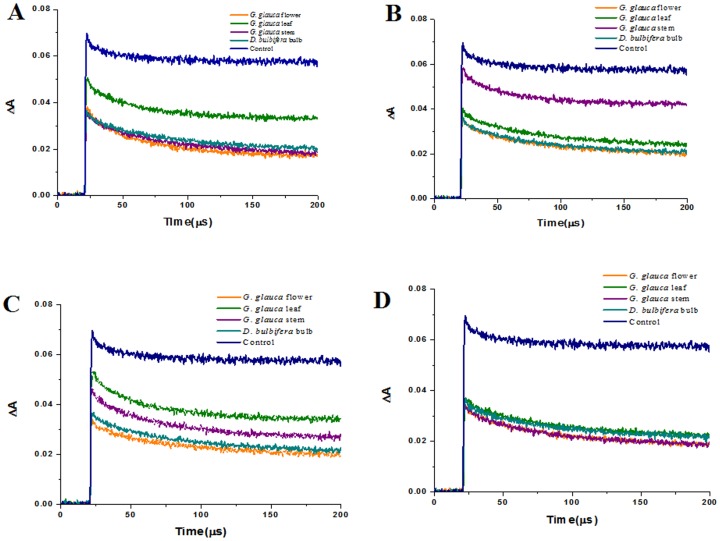
Kinetic decay of pulse radiolysis generated ABTS^•+^ radical by plant extracts. Petroleum ether extracts, (B) Ethyl acetate extracts, (C) Methanolic extracts and (D) Ethanolic (70% v/v) extracts.

Pulse radiolysis studies provided a significant insight to ABTS^•+^ radical scavenging potential of the extracts tested (Figure 2 and 3). Linear plot of pseudo-first order rate constant (K_abs_) was used to extrapolate the second order decay constants with kinetic processor software. Petroleum ether extract of *G. glauca* leaf (1.81×10^6^) showed highest activity while ascorbic acid failed to show any activity. Ethyl acetate extract of *D. bulbifera* (2.33×10^6^) showed an enhanced activity. Methanolic extracts of *G. glauca* stem (1.72×10^6^) and flower (1.60×10^6^) were found to be superior as compared to leaf (1.24×10^6^) and *D. bulbifera* bulb (1.42×10^6^). 70% (v/v) ethanolic extracts of *G. glauca* leaf (1.34×10^6^) and *D. bulbifera* bulb (1.31×10^6^) showed an identical level of pulse radiolysis generated ABTS^•+^ radical scavenging activity. Similarly, *G. glauca* flower (1.12×10^6^) and stem (1.19×10^6^) showed almost identical activity.

### DPPH radical scavenging activity

Petroleum ether extracts of leaf and flower of *G. glauca* and bulb of *D. bulbifera* showed comparable activity in a range between 60 to 65% (Table 4). *D. bulbifera* bulb showed slightly lesser activity in all extracts as compared to *G. glauca*. *G. glauca* leaf showed highest activity in both methanolic and ethanolic extracts (*P*<0.05) as compared to ascorbic acid. Stem and flower showed DPPH scavenging activity in a comparable range in case of both methanolic and 70% (v/v) ethanolic extracts.

**Table 4:** DPPH radical scavenging activity by plant extracts.

Plant extract	% DPPH radical scavenging activity			
AA = 87.44±0.45	Petroleum ether	Ethyl acetate	Methanol	Ethanol (70% v/v)
*G.glauca*				
Flower	62.36±4.97	88.70±0.31	92.47±0.62*	88.17±0.62
Leaf	63.44±1.24	94.62±1.24	95.16±0.93*	92.47±0.62*
Stem	53.25±0.31	89.78±4.66	90.32±2.48*	88.70±0.93
*D.bulbifera*				
Bulb	61.82±1.55	82.79±1.24	84.94±0.62*	80.64±1.24

AA = Ascorbic acid; the data is indicated as the mean ± SEM; [*n = *3]. Data with different asterisks (*) shows significant difference (*P*<0.05), two-tailed student t-test.

### Ferric reducing antioxidant power

**Figure 4:**
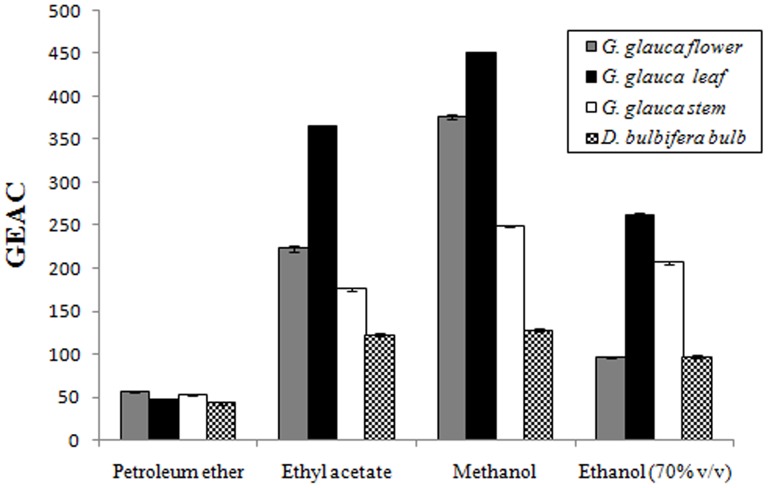
Ferric reducing antioxidant power of plant extracts.

**Figure 5:**
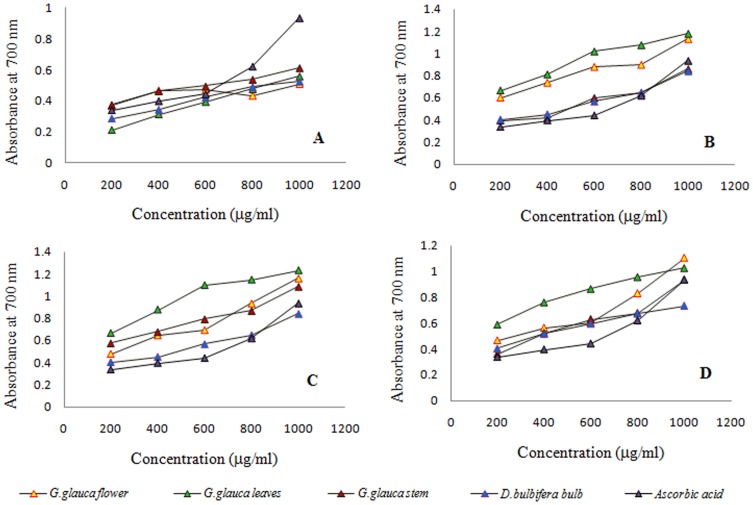
Reducing activity of plant extracts. (A) Petroleum ether extract, (B) Ethyl acetate extract, (C) Methanol extract, (D) Ethanol (70% v/v) of plants at various concentrations.

Petroleum ether extracts of *G.glauca* flower, leaf, stem and bulb of *D. bulbifera* exhibited an activity in a range of 40 to 60 GAEAC. *G. glauca* leaf showed highest GAEAC values in all extracts. In case of ethyl acetate extracts leaf showed 364.7±2.99 GAEAC followed by flower, stem and *D. bulbifera* bulb showing 223.03±2.99, 176.06±2.12 and 123.03±1.9 GAEAC respectively (Figure 4). Methanolic extracts of *G. glauca* leaf showed highest GAEAC equivalent to 451.21±0.66 followed by flower, stem and *D. bulbifera* bulb. However ascorbic acid (1304±1.33) showed a very high activity. 70% (v/v) ethanolic extracts showed ferric reducing capacity intermediate between ethyl acetate and methanol extracts. *D. bulbifera* bulb (97.88±2.11 GAEAC) showed an identical activity to *G. glauca* flower. In case of concentration dependent reducing power, it was observed that petroleum ether extract of *G. glauca* stem showed excellent reducing power as compared to *G. glauca* leaf and *D. bulbifera* bulb which showed a moderate activity (Figure 5). However, in case of other extracts *G. glauca* leaf showed superior reducing power. Ethyl acetate extract of *G. glauca* flower exhibited stronger reducing activity in comparison with both *G. glauca* stem and *D. bulbifera* bulb. Among ethanolic extracts, *G. glauca* leaf showed highest activity. Although *G. glauca* stem, flower and *D. bulbifera* bulb exhibited identical reducing activity at concentration between 200 to 600 µg/mL. Aat higher concentration *G. glauca* flower showed better reducing power.

### Superoxide anion scavenging activity

Highest superoxide anion scavenging activity was exhibited by methanolic extracts (Table 5). Petroleum ether extract of *D. bulbifera* bulb showed highest activity while *G. glauca* showed least. Ethyl acetate extract of *G. glauca* leaf showed highest activity. Methanol extracts of both *G.glauca* stem and *D. bulbifera* bulb showed almost identical activity, while *G. glauca* leaf showed highest (*P*<0.05). 70% (v/v) ethanolic extracts of *G. glauca* leaf exhibited excellent superoxide anion scavenging activity while *D.bubifera* showed an activity almost equivalent to ascorbic acid.

**Table 5:** Superoxide anion scavenging activity of plant extracts.

Plant extract	% Superoxide anion scavenging activity			
AA = 55.07±1.83	Petroleum ether	Ethyl acetate	Methanol	Ethanol (70% v/v)
*G.glauca*				
Flower	14.06±1.44	61.67±1.71	67.51±0.23*	59.75±0.62
Leaf	22.96±2.07	64.52±0.70	72.12±0.53*	63.06±1.42
Stem	22.43±1.50	50.38±1.00	60.45±0.43*	58.37±1.00
*D.bulbifera*				
Bulb	26.88±1.28	57.60±0.81	59.75±0.98*	54.76±1.20

AA = Ascorbic acid; the data is indicated as the mean ± SEM; [*n = *3]. Data with asterisk (*) shows significant difference (*P*<0.05), two-tailed student t-test.

In the alternative method, *G. glauca* stem showed better activity among petroleum ether extracts (Table 6). However, in rest of extracts *G. glauca* leaf was found to exhibit excellent activity, highest being methanolic extract (*P*<0.05).

**Table 6:** Superoxide radical scavenging activity of plant extracts.

Plant extract	% Superoxide radical scavenging activity			
AA = 61.44±0.7	Petroleum ether	Ethyl acetate	Methanol	Ethanol (70% v/v)
*G.glauca*				
Flower	29.46±1.55	66.42±0.44	72.44±1.02*	64.27±1.41
Leaf	33.25±1.33	69.39±0.79	73.35±0.72*	63.53±1.49
Stem	39.03±0.97	57.26±1.22	63.61±0.76*	60.23±1.16
*D.bulbifera*				
Bulb	28.30±0.36	59.24±1.44	59.65±1.41*	57.34±1.41

AA = Ascorbic acid; the data is indicated as the mean ± SEM; [*n = *3]. Data with asterisk (*) shows significant difference (*P*<0.05), two-tailed student t-test.

### Nitric oxide scavenging activity

*G. glauca* as well as *D. bulbifera* exhibited high activity with methanol extract while both ethyl acetate and 70% (v/v) ethanol extract showed moderate activity, least being petroleum ether extracts (Table 7). Methanol extracts of both *G. glauca* flower and leaf exhibited identically high activity (*P*<0.05) indicating their excellent activity. Ethanol extracts showed a moderate nitric oxide scavenging activity.

**Table 7:** Nitric oxide scavenging activity of plant extracts.

Plant extract	% Nitric oxide scavenging activity			
AA = 23.21±1.54	Petroleum ether	Ethyl acetate	Methanol	Ethanol (70% v/v)
*G.glauca*				
Flower	22.86±0.47	62.71±0.62	68.10±0.42*	60.87±0.09
Leaf	26.51±0.36	65.85±0.45	68.85±0.24*	61.58±0.61
Stem	23.17±0.48	50.80±0.18	63.66±0.74*	57.59±0.84
*D.bulbifera*				
Bulb	20.57±0.57	54.55±0.21	57.59±0.64*	49.85±0.16

AA = Ascorbic acid; the data is indicated as the mean ± SEM; [*n = *3]. Data with asterisk (*) shows significant difference (*P*<0.05), two-tailed student t-test.

**Figure 6:**
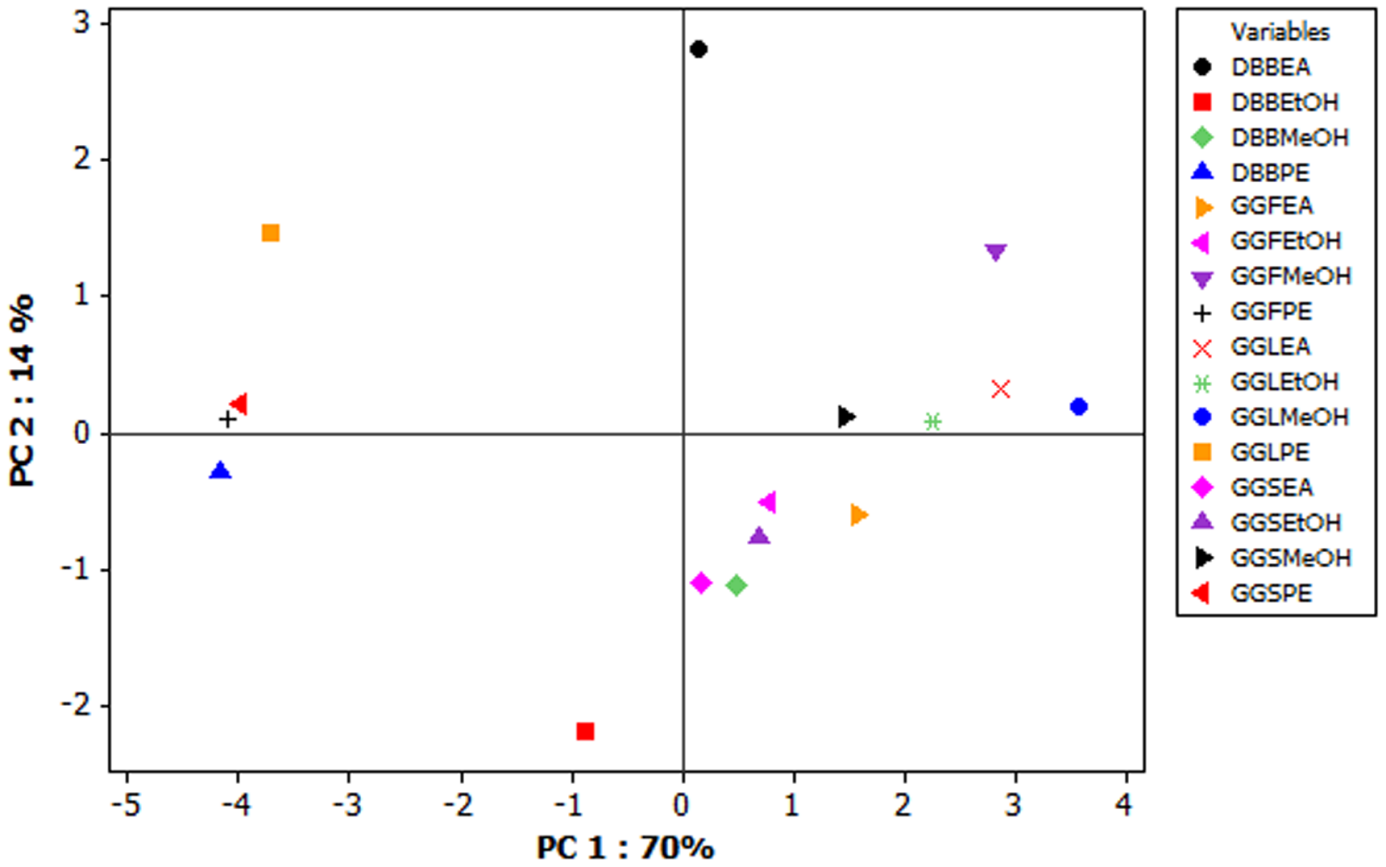
Principal component analysis of antioxidant activity represented by scatter plot. GGF - *G. glauca* flower; GGL - *G. glauca* leaf; GGS – *G. glauca* stem; DBB – *D. bulbifera* bulbs; PE: Petroleum ether extract; EA: Ethyl acetate extract; MeOH: Methanol extract; EtOH: Ethanol extract (70%).

Principal component analysis was used to evaluate the antioxidant activity and the various solvent extracts. The first two components (PC1 and 2) explained 84% of the total variance (Figure 6). PCA separated the samples by antioxidant activity (PC 1) and different extracts (on the basis of solvents). In the left quadrant all the petroleum ether extracts got grouped indicating the similar behavioral pattern of lower antioxidant activity. Similarly, on the right quadrants all of the other extracts were grouped showing a comparatively superior activity, methanol being the most potent. Thus PCA results were in correlation with that of our observed experimental data.

### HPTLC fingerprint profile

**Figure 7:**
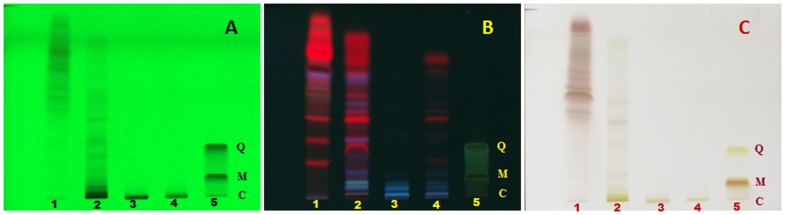
HPTLC fingerprint of extracts of *G. glauca* flower. (1) Petroleum ether extract, (2) Ethyl acetate extract, (3) Methanol extract, (4) Ethanol extract (70% v/v) at (A) 254 nm, (B) 366 nm and (C) 600 nm. Standards used are quercetin (Q), myricetin (M) and catechin (C) in lane 5.

HPTLC fingerprint of *G. glauca* flower exhibited maximum number of twelve compounds at 254 nm in methanolic extract (Figure 7). Compounds with *Rf* values of 0.74 and 0.78 was found to be common in both petroleum ether and 70% ethanolic extracts. At 366 nm as well, number of bands were found to be maximum in methanolic extract of *G. glauca* flower. Band at *Rf*: 0.78 was found to be present in both petroleum ether and ethyl acetate extracts. Similarly, bands at *Rf*: 0.16, 0.31, and 0.59 were observed in both ethyl acetate and methanolic extracts while bands at *Rf*: 0.16 and 0.82 were found in methanolic as well as 70% (v/v) ethanolic extracts. In case of 600 nm, band corresponding to *Rf*: 0.37 was present in both petroleum ether extract and 70% (v/v) ethanolic extracts.

**Figure 8:**
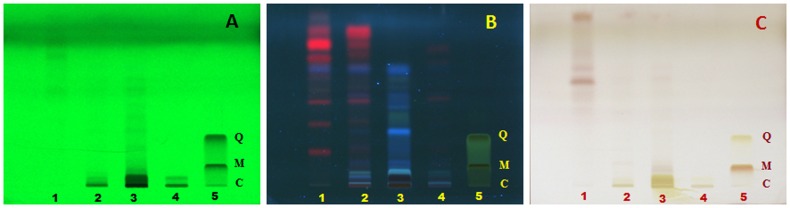
HPTLC fingerprint of extracts of *G. glauca* leaf. (1) Petroleum ether extract, (2) Ethyl acetate extract, (3) Methanol extract, (4) Ethanol extract (70% v/v) at (A) 254 nm, (B) 366 nm and (C) 600 nm. Standards used are quercetin (Q), myricetin (M) and catechin (C) in lane 5.

In case of *G. glauca* leaf, ethyl acetate extract showed a maximum of 11 and 12 band at 254 nm and 366 nm respectively (Figure 8). The band at 254 nm corresponding to *Rf*: 0.78 was present in all extracts except petroleum ether while the band at 366 nm at *Rf*: 0.54 was found to be common for petroleum ether and 70% (v/v) ethanolic extracts. Maximum number of bands after derivatisation were visible at 600 nm in ethyl acetate extract followed by petroleum ether extract.

**Figure 9:**
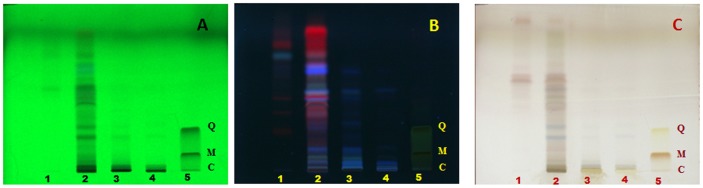
HPTLC fingerprint of extracts of *G. glauca* stem. (1) Petroleum ether extract, (2) Ethyl acetate extract, (3) Methanol extract, (4) Ethanol extract (70% v/v) at (A) 254 nm, (B) 366 nm and (C) 600 nm. Standards used are quercetin (Q), myricetin (M) and catechin (C) in lane 5.

A similar trend was observed for ethyl acetate extract of *G. glauca* stem showing 10, 14 and 13 distinct bands at 254 nm, 366 nm and 600 nm respectively (Figure 9). The band visible at 254 nm at *Rf*: 0.56 was present in all extracts except 70% (v/v) ethanolic extract while one at *Rf*: 0.46 was found in particularly petroleum ether and ethyl acetate. However, the band at *Rf*: 0.31 was found only in petroleum ether and 70% (v/v) ethanolic extracts. In case of bands visible at 366 nm, the band at *Rf*: 0.32 and 0.73 were found in both petroleum ether and ethyl acetate extracts while the band at *Rf*: 0.45 was common to both methanolic and 70% (v/v) ethanolic extracts. After derivatisation the band visible in 600 nm at *Rf*: 0.42 was found to be most prominent in all extracts of *G. glauca* stem. The band at *Rf*: 0.87 was found to be present in both petroleum ether and ethyl acetate extracts.

**Figure 10:**
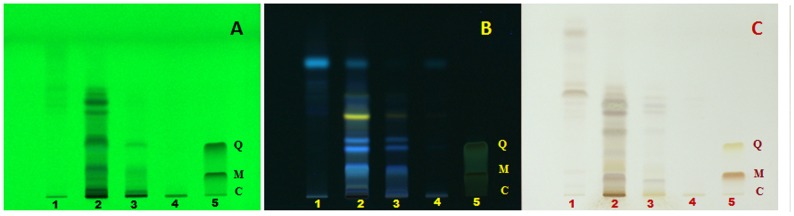
HPTLC fingerprint of extracts of bulbs of *D. bulbifera.* (1) Petroleum ether extract, (2) Ethyl acetate extract, (3) Methanol extract, (4) Ethanol extract (70% v/v) at (A) 254 nm, (B) 366 nm and (C) 600 nm. Standards used are quercetin (Q), myricetin (M) and catechin (C) in lane 5.

HPTLC fingerprint showed a maximum of 12 bands at 254 nm in petroleum ether extract of *D. bulbifera* bulb (Figure 10). The band at *Rf*: 0.41 was present in both petroleum ether and ethyl acetate extracts while one at *Rf*: 0.79 was present in all extracts except petroleum ether. A band at 366 nm corresponding to *Rf*: 0.66 was found in both ethyl acetate and 70% (v/v) ethanol extracts. Similarly, band visible at 600 nm at *Rf*: 0.48 was present in both methanolic and 70% (v/v) ethanolic extracts.

### GC-TOF-MS analyses

Major phytochemical among the identified compounds in petroleum ether extracts of *G. glauca* flower leaf and stem was diphenyl sulfone (C_12_H_10_O_2_S) with RT 725.5 (s) and a peak area percentage of 57.55 (Table 8). Second major phytochemical identified was n-hexadecanoic acid (C_16_H_32_O_2_) with RT 710.1 (s) and a peak are percentage of 4.81. Similarly, diphenyl sulfone and n-hexadecanoic acid were found to be major phytochemicals present in petroleum ether extracts of *G. glauca* leaf and stem as well. In case of petroleum ether extract of *D. bulbifera* bulbs, among identified compounds, ethyl ester of undecanoic acid (C_13_H_26_O_2_), Z-1,9-dodecadiene (C_12_H_22_) and n-hexadecanoic acid were found to be predominant. In case of ethyl acetate extracts of *G. glauca*, diphenyl sulfone and methyl ester of octadecanoic acid (C_19_H_38_O_2_) was detected in higher proportion. However, pentadecanoic acid, 14-methyl-, methyl ester (C_17_H_34_O_2)_ was also present in significant level in *G. glauca* stem. Notable amount of diosgenin (C_27_H_42_O_3_) with RT 926.5(s) and peak area percentage of 94.05 was confirmed in ethyl acetate extract of *D. bulbifera* bulbs. Apart from diosgenin, ethyl ester of eicosanoic acid (C_22_H_44_O_2_) was also found. Methanol extracts also exhibited higher amount of diphenyl sulfone and octadecanoic acid in all three parts of *G. glauca*, highest being in flower while for 70% (v/v) ethanolic extract *G. glauca* stem showed highest diphenyl sulfone content. Diosgenin was prevalent as major phytochemical even in 70% (v/v) ethanolic extract of *D. bulbifera.*

**Table 8:** Main compounds detected by GC-TOF-MS.

Extracts	Name of the compound	Formula	Molecular mass	Area %	Retention time (s)	
**Petroleum ether**						
*G. glauca* flower	Phenol, 2,4-bis(1,1-dimethylethyl)-	C_14_H_22_O	206.17	2.33	557.2	
	Diethyl phthalate	C_12_H_14_O_4_	222.09	1.32	589.6	
	Dodecyl acrylate	C_15_H_28_O_2_	240.21	3.01	616	
	Hexadecane	C_16_H_34_	226.27	2.03	617.2	
	1,2-Benzenedicarboxylic acid, bis(2-methylpropyl) ester	C_16_H_22_O_4_	278.15	1.45	684.9	
	Pentadecanoic acid, 14-methyl-, methyl ester	C_17_H_34_O_2_	270.26	1.6	697.2	
	n-Hexadecanoic acid	C_16_H_32_O_2_	256.24	4.81	710.1	
	Dibutyl phthalate	C_16_H_22_O_4_	278.15	2.01	720.1	
	Diphenyl sulfone	C_12_H_10_O_2_S	218.04	57.55	725.5	
	1,6-Heptadiene, 2-methyl-6-phenyl-	C_14_H_18_	186.14	3.09	738.3	
	Pyridazin-3(2H)-one, 2-cyclohexyl-5-hydroxy-4-methoxy-	C_11_H_16_N_2_O_3_	224.12	1.29	763.9	
	Tridecanoic acid, methyl ester	C_14_H_28_O_2_	228.21	3.28	777	
	Hexadecanoic acid, 2-hydroxy-1-(hydroxymethyl)ethyl ester	C_19_H_38_O_4_	330.28	1.92	1023.2	
*G. glauca* leaf	Phenol, 2,4-bis(1,1-dimethylethyl)-	C_14_H_22_O	206.17	1.4	556.4	
	Dodecyl acrylate	C_15_H_28_O_2_	240.21	6.85	615.4	
	Pentadecanoic acid, 14-methyl-, methyl ester	C_17_H_34_O_2_	270.26	3.95	696.9	
	n-Hexadecanoic acid	C_16_H_32_O_2_	256.24	18.72	711.8	
	1,2-Benzenedicarboxylic acid, butyl 2-ethylhexyl ester	C_20_H_30_O_4_	334.21	1.51	720	
	Diphenyl sulfone	C_12_H_10_O_2_S	218.04	30.34	725.2	
	1,6-Heptadiene, 2-methyl-6-phenyl-	C_14_H_18_	186.14	1.61	761.6	
	Phytol	C_20_H_40_O	296.31	2.34	775.7	
	Octadecanoic acid, methyl ester	C_19_H_38_O_2_	298.29	7.57	776.9	
	9,12,15-Octadecatrienoic acid, (Z,Z,Z)-	C_18_H_30_O_2_	278.22	4.61	789.7	
	Benzene, (1,2-dicyclopropyl-2-phenylethyl)-	C_20_H_22_	262.17	1.56	809.8	
	Benzene, (1,2-dicyclopropyl-2-phenylethyl)-	C_20_H_22_	262.17	1	823.3	
	1-Phenylcyclopentanecarboxylic acid	C_12_H_14_O_2_	190.1	1.09	879.5	
*G. glauca* stem	Phenol, 3,5-bis(1,1-dimethylethyl)-	C_14_H_22_O	206.17	1.72	556.8	
	Dodecyl acrylate	C_15_H_28_O_2_	240.21	5.21	615.6	
	n-Hexadecanoic acid	C_16_H_32_O_2_	256.24	9.48	710.4	
	Dibutyl phthalate	C_16_H_22_O_4_	278.15	1.37	719.7	
	Diphenyl sulfone	C_12_H_10_O_2_S	218.04	52.7	724.9	
	1,6-Heptadiene, 2-methyl-6-phenyl-	C_14_H_18_	186.14	5.88	738	
	1,6-Heptadiene, 2-methyl-6-phenyl-	C_14_H_18_	186.14	1.07	761.2	
	1H-Indene, 1-methyl-3-propyl-	C_13_H_16_	172.13	2.93	763.7	
	17-Octadecynoic acid	C_18_H_32_O_2_	280.24	1.94	785.2	
	Benzene, (1,2-dicyclopropyl-2-phenylethyl)-	C_20_H_22_	262.17	1.08	809.2	
	Hexadecanoic acid, 2-hydroxy-1-(hydroxymethyl)ethyl ester	C_19_H_38_O_4_	330.28	1.39	1022.4	
*D. bulbifera* bulb	3-Oxo-androsta-1,4-dien-17á-spiro-2'-3'-oxo-oxetane	C_21_H_26_O_3_	326.19	1.17	599.7	
	Apiol	C_12_H_14_O_4_	222.09	1.44	602.1	
	1,2-Benzenedicarboxylic acid, butyl 8-methylnonyl ester	C_22_H_34_O_4_	362.25	1.13	685.1	
	Pentadecanoic acid, 14-methyl-, methyl ester	C_17_H_34_O_2_	270.26	2.3	697.3	
	n-Hexadecanoic acid	C_16_H_32_O_2_	256.24	16.74	712.5	
	Undecanoic acid, ethyl ester	C_13_H_26_O_2_	214.19	27.94	722.8	
	9,12-Octadecadienoic acid, methyl ester, (E,E)-	C_19_H_34_O_2_	294.26	1.04	767.7	
	Z-1,9-Dodecadiene	C_12_H_22_	166.17	10.61	798.6	
	9-Octadecenoic acid, ethyl ester	C_20_H_38_O_2_	310.29	4.61	799.9	
	Methyl 17-methyl-octadecanoate	C_20_H_40_O_2_	312.3	4.87	808.1	
	2,4-Hexadienedioic acid, 3,4-diethyl-, dimethyl ester, (Z,Z)-	C_12_H_18_O_4_	226.12	7.49	916.5	
	Benzene, pentafluoro[(2-methylphenoxy)methyl]-	C_14_H_9_F_5_O	288.06	6.42	921.6	
	3-Methoxyestra-1,3,5(10),8,14-pentaen-17-one (+,–)-	C_19_H_20_O_2_	280.15	2.29	1023	
	Octadecanoic acid, ethyl ester	C_20_H_40_O_2_	312.3	1.18	1093.3	
**Ethyl acetate**						
*G. glauca* flower	Tetradecane	C_14_H_30_	198.23	1.01	511.2	
	Benzoic acid, 4-ethoxy-, ethyl ester	C_11_H_14_O_3_	194.09	1.44	563.5	
	Diethyl phthalate	C_12_H_14_O_4_	222.09	1.98	588.7	
	Dodecyl acrylate	C_15_H_28_O_2_	240.21	5.49	615.4	
	3,5-Dimethyldodecane	C_14_H_30_	198.23	2.93	616.6	
	Hexadecane	C_16_H_34_	226.27	1.02	651.5	
	1,2-Benzenedicarboxylic acid, bis(2-methylpropyl) ester	C_16_H_22_O_4_	278.15	1.42	684.6	
	Hexadecanoic acid, methyl ester	C_17_H_34_O_2_	270.26	2.87	697	
	Heptyl methyl ethylphosphonate	C_10_H_23_O_3_P	222.14	1.12	724.3	
	Diphenyl sulfone	C_12_H_10_O_2_S	218.04	36.29	725.3	
	1,6-Heptadiene, 2-methyl-6-phenyl-	C_14_H_18_	186.14	5.71	738	
	Squalene	C_30_H_50_	410.39	7.2	759.6	
	2(1H)-Naphthalenone, octahydro-4a-phenyl-, trans-	C_16_H_20_O	228.15	1.21	761.4	
	1H-Indene, 1-methyl-3-propyl-	C_13_H_16_	172.13	2.64	763.8	
	Octadecanoic acid, methyl ester	C_19_H_38_O_2_	298.29	5.45	776.9	
	Benzene, (1,2-dicyclopropyl-2-phenylethyl)-	C_20_H_22_	262.17	1.09	809.5	
	Benzyl á-d-glucoside	C_13_H_18_O_6_	270.11	2.42	861.8	
	Hexadecanoic acid, 2-hydroxy-1-(hydroxymethyl)ethyl ester	C_19_H_38_O_4_	330.28	1.66	1023.2	
*G. glauca* leaf	Undecane	C_11_H_24_	156.19	1.03	511.5	
	D-Galactose, 6-deoxy-	C_6_H_12_O_5_	164.07	4.2	529.4	
	Diethyl Phthalate	C_12_H_14_O_4_	222.09	1.67	588.8	
	Dodecyl acrylate	C_15_H_28_O_2_	240.21	9.47	615.3	
	Hexadecane	C_16_H_34_	226.27	2.8	616.1	
	Phthalic acid, isobutyl nonyl ester	C_21_H_32_O_4_	348.23	1.12	684.6	
	Hexadecane	C_16_H_34_	226.27	2.18	686.5	
	Pentadecanoic acid, 14-methyl-, methyl ester	C_17_H_34_O_2_	270.26	4.97	697	
	Dibutyl phthalate	C_16_H_22_O_4_	278.15	1.55	719.8	
	Diphenyl sulfone	C_12_H_10_O_2_S	218.04	25.37	725.4	
	1,6-Heptadiene, 2-methyl-6-phenyl-	C_14_H_18_	186.14	1.12	736.8	
	Squalene	C_30_H_50_	410.39	5.64	760	
	1,6-Heptadiene, 2-methyl-6-phenyl-	C_14_H_18_	186.14	2.11	761.5	
	Benzene, (1,1-dimethyl-2-butynyl)-	C_12_H_14_	158.11	4.61	763.8	
	Octadecanoic acid, methyl ester	C_19_H_38_O_2_	298.29	9.48	776.9	
	Benzene, (1,2-dicyclopropyl-2-phenylethyl)-	C_20_H_22_	262.17	1.8	809.5	
	Benzene, (1,2-dicyclopropyl-2-phenylethyl)-	C_20_H_22_	262.17	1.14	823	
	1-Phenylcyclopentanecarboxylic acid	C_12_H_14_O_2_	190.1	1.32	879.3	
	1-Iodo-2-methylundecane	C_12_H_25_I	296.1	2.24	879.9	
	Hexadecanoic acid, 2-hydroxy-1-(hydroxymethyl)ethyl ester	C_19_H_38_O_4_	330.28	1.02	1022.8	
*G. glauca* stem	Decane, 5,6-dimethyl-	C_12_H_26_	170.2	1.21	512.1	
	Benzaldehyde, 3-hydroxy-4-methoxy-	C_8_H_8_O_3_	152.05	1.17	525.8	
	Benzoic acid, 4-ethoxy-, ethyl ester	C_11_H_14_O_3_	194.09	1.01	564.7	
	1-Docosene	C_22_H_44_	308.34	1.48	580.4	
	Diethyl phthalate	C_12_H_14_O_4_	222.09	2.02	589.8	
	Dodecyl acrylate	C_15_H_28_O_2_	240.21	6.36	616.1	
	Decane, 2,6,6-trimethyl-	C_13_H_28_	184.22	2.82	617.1	
	1,2-Benzenedicarboxylic acid, bis(2-methylpropyl) ester	C_16_H_22_O_4_	278.15	1.82	685.1	
	Pentadecanoic acid, 14-methyl-, methyl ester	C_17_H_34_O_2_	270.26	10.81	697.4	
	n-Hexadecanoic acid	C_16_H_32_O_2_	256.24	16.29	711.2	
	Dibutyl phthalate	C_16_H_22_O_4_	278.15	3.56	720.4	
	Diphenyl sulfone	C_12_H_10_O_2_S	218.04	10.22	725.7	
	1,6-Heptadiene, 2-methyl-6-phenyl-	C_14_H_18_	186.14	6.55	738.6	
	1,6-Heptadiene, 2-methyl-6-phenyl-	C_14_H_18_	186.14	1.27	761.8	
	Pyridazin-3(2H)-one, 2-cyclohexyl-5-hydroxy-4-methoxy-	C_11_H_16_N_2_O_3_	224.12	2.82	764.3	
	Phytol	C_20_H_40_O	296.31	1.59	775.9	
	Octadecanoic acid, methyl ester	C_19_H_38_O_2_	298.29	7.58	777.2	
	Z-1,9-Hexadecadiene	C_16_H_30_	222.23	1.11	786.1	
	cis-3-Butyl-4-vinyl-cyclopentene	C_11_H_18_	150.14	1.15	788.9	
	Benzene, (1,2-dicyclopropyl-2-phenylethyl)-	C_20_H_22_	262.17	1.23	809.9	
	Hexadecanoic acid, 2-hydroxy-1-(hydroxymethyl)ethyl ester	C_19_H_38_O_4_	330.28	1.42	1023.1	
*D. bulbifera* bulb	Eicosanoic acid, ethyl ester	C_22_H_44_O_2_	340.33	1.03	681	
	Diosgenin	C_27_H_42_O_3_	414.31	94.05	926.5	
**Methanol**						
*G. glauca* flower	5-[1], [2], [4]Triazol-1-yl-pyrrolidin-2-one	C_6_H_8_N_4_O	152.07	1.97	510.5	
	Diethyl Phthalate	C_12_H_14_O_4_	222.09	1.52	589.2	
	Dodecyl acrylate	C_15_H_28_O_2_	240.21	5.07	615.7	
	Hexadecane	C_16_H_34_	226.27	2.13	616.8	
	Phthalic acid, cyclobutyl hexyl ester	C_18_H_24_O_4_	304.17	1.09	684.8	
	Pentadecanoic acid, 14-methyl-, methyl ester	C_17_H_34_O_2_	270.26	2.89	697.1	
	Diphenyl sulfone	C_12_H_10_O_2_S	218.04	54.82	725.5	
	1,6-Heptadiene, 2-methyl-6-phenyl-	C_14_H_18_	186.14	5.6	738.2	
	1,6-Heptadiene, 2-methyl-6-phenyl-	C_14_H_18_	186.14	1.09	761.4	
	Octadecanoic acid, methyl ester	C_19_H_38_O_2_	298.29	5.43	776.9	
	Hexadecanoic acid, 2-hydroxy-1-(hydroxymethyl)ethyl ester	C_19_H_38_O_4_	330.28	2.14	1022.8	
*G. glauca* leaf	1-Undecene, 5-methyl-	C_12_H_24_	168.19	1.77	579.6	
	Diethyl phthalate	C_12_H_14_O_4_	222.09	1.41	589	
	Dodecyl acrylate	C_15_H_28_O_2_	240.21	7.51	615.6	
	3-Hexanone, 2,4-dimethyl-	C_8_H_16_O	128.12	2.62	616.3	
	Diphenyl sulfone	C_12_H_10_O_2_S	218.04	10.28	646.1	
	1,2-Benzenedicarboxylic acid, bis(2-methylpropyl) ester	C_16_H_22_O_4_	278.15	1.08	684.6	
	Pentadecanoic acid, 14-methyl-, methyl ester	C_17_H_34_O_2_	270.26	3.34	697.1	
	Diphenyl sulfone	C_12_H_10_O_2_S	218.04	1.3	724.2	
	Diphenyl sulfone	C_12_H_10_O_2_S	218.04	42.49	725.7	
	1,6-Heptadiene, 2-methyl-6-phenyl-	C_14_H_18_	186.14	1.78	761.3	
	Octadecanoic acid, methyl ester	C_19_H_38_O_2_	298.29	7.72	776.8	
	Benzene, (1,2-dicyclopropyl-2-phenylethyl)-	C_20_H_22_	262.17	1.75	809.2	
	Benzene, (1,2-dicyclopropyl-2-phenylethyl)-	C_20_H_22_	262.17	1.17	822.8	
	1-Phenylcyclopentanecarboxylic acid	C_12_H_14_O_2_	190.1	1.47	879.1	
*G. glauca* stem	Hexadecane	C_16_H_34_	226.27	1.15	509.8	
	Benzoic acid, 4-ethoxy-, ethyl ester	C_11_H_14_O_3_	194.09	1.11	562.6	
	Diethyl phthalate	C_12_H_14_O_4_	222.09	2.27	588	
	Dodecyl acrylate	C_15_H_28_O_2_	240.21	5.74	614.8	
	1,2-Benzenedicarboxylic acid, bis(2-methylpropyl) ester	C_16_H_22_O_4_	278.15	1.1	684.3	
	Pentadecanoic acid, 14-methyl-, methyl ester	C_17_H_34_O_2_	270.26	2.55	696.8	
	Diphenyl sulfone	C_12_H_10_O_2_S	218.04	52.49	725	
	5,8,11-Heptadecatriynoic acid, methyl ester	C_18_H_24_O_2_	272.18	5.52	737.8	
	1,6-Heptadiene, 2-methyl-6-phenyl-	C_14_H_18_	186.14	1.1	761.3	
	Trimethadione	C_6_H_9_NO_3_	143.06	2.4	763.5	
	Octadecanoic acid, methyl ester	C_19_H_38_O_2_	298.29	5.2	776.7	
	Hexadecanoic acid, 2-hydroxy-1-(hydroxymethyl)ethyl ester	C_19_H_38_O_4_	330.28	1.31	1023	
*D. bulbifera* bulb	2-Pyrrolidinone, 1-methyl-	C_5_H_9_NO	99.07	17.83	420.3	
	2-Pentanol, acetate	C_7_H_14_O_2_	130.1	4.59	475.5	
	Butylated hydroxytoluene	C_15_H_24_O	220.18	16.19	554.1	
	9H-Fluorene, 9-methylene-	C_14_H_10_	178.08	10.23	675.9	
	5-(Methylamino)-1,2,3,4-thiatriazole	C_2_H_4_N_4_S	116.02	5.28	721.6	
	Acetic acid, [(1,1-dimethylethyl)thio]-	C_6_H_12_O_2_S	148.06	10.87	754.8	
	2-(1-Methylcyclopentyloxy)-tetrahydropyran	C_11_H_20_O_2_	184.15	5.24	808.4	
	Pentanoic acid, 1,1-dimethylpropyl ester	C_10_H_20_O_2_	172.15	6.09	809	
	Decane, 2,4,6-trimethyl-	C_13_H_28_	184.22	8.62	828.5	
	Undecane	C_11_H_24_	156.19	3.09	861.3	
	Heptylcyclohexane	C_13_H_26_	182.2	4.49	862.4	
**Ethanol (70%)**						
*G. glauca* flower	Tetradecane	C_14_H_30_	198.23	1.18	512.9	
	Benzoic acid, 4-ethoxy-, ethyl ester	C_11_H_14_O_3_	194.09	1.22	564.9	
	Diethyl phthalate	C_12_H_14_O_4_	222.09	1.92	589.8	
	Dodecyl acrylate	C_15_H_28_O_2_	240.21	6.62	616	
	1,2-Benzenedicarboxylic acid, bis(2-methylpropyl) ester	C_16_H_22_O_4_	278.15	1.51	684.8	
	Pentadecanoic acid, 14-methyl-, methyl ester	C_17_H_34_O_2_	270.26	3.13	697.1	
	Dibutyl phthalate	C_16_H_22_O_4_	278.15	1.06	719.8	
	Diphenyl sulfone	C_12_H_10_O_2_S	218.04	1.45	724.4	
	Diphenyl sulfone	C_12_H_10_O_2_S	218.04	43.87	725.4	
	5,8,11-Heptadecatriynoic acid, methyl ester	C_18_H_24_O_2_	272.18	7.7	738.1	
	1,6-Heptadiene, 2-methyl-6-phenyl-	C_14_H_18_	186.14	1.54	761.3	
	1H-Indene, 1-methyl-3-propyl-	C_13_H_16_	172.13	3.39	763.7	
	Octadecanoic acid, methyl ester	C_19_H_38_O_2_	298.29	6.41	776.8	
	Benzene, (1,2-dicyclopropyl-2-phenylethyl)-	C_20_H_2_2	262.17	1.35	809.3	
	(4-Isopropylidenebicyclo[3.2.0]hept-2-en-6-ylidene)acetic acid, methyl ester	C_13_H_16_O_2_	204.12	1.04	879	
	Hexadecanoic acid, 2-hydroxy-1-(hydroxymethyl)ethyl ester	C_19_H_38_O_4_	330.28	1.4	1022.4	
*G. glauca* leaf	Decane, 2,5,9-trimethyl-	C_13_H_28_	184.22	1.17	510	
	Benzoic acid, 4-ethoxy-, ethyl ester	C_11_H_14_O_3_	194.09	1	562.9	
	Octadecanoic acid, 2-hydroxy-1-(hydroxymethyl)ethyl ester	C_21_H_42_O_4_	358.31	1.56	570	
	Dodecyl acrylate	C_15_H_28_O_2_	240.21	6.63	615	
	Hexadecane	C_16_H_34_	226.27	3.04	616.2	
	Diphenyl sulfone	C_12_H_10_O_2_S	218.04	57.24	724.8	
	5,8,11-Heptadecatriynoic acid, methyl ester	C_18_H_24_O_2_	272.18	6.21	738	
	1,6-Heptadiene, 2-methyl-6-phenyl-	C_14_H_18_	186.14	1.19	761.3	
	1H-Indene, 1-methyl-3-propyl-	C_13_H_16_	172.13	1.17	763	
	Benzene, (1,2-dicyclopropyl-2-phenylethyl)-	C_20_H_22_	262.17	1.14	809.3	
	Hexadecanoic acid, 2-hydroxy-1-(hydroxymethyl)ethyl ester	C_19_H_38_O_4_	330.28	1.58	1023.4	
*G. glauca* stem	Hexadecane	C_16_H_34_	226.27	1.29	511.6	
	Benzoic acid, 4-ethoxy-, ethyl ester	C_11_H_14_O_3_	194.09	1.05	563.9	
	Decane, 3,7-dimethyl-	C_12_H_26_	170.2	3.43	582.2	
	Diethyl phthalate	C_12_H_14_O_4_	222.09	2.39	589	
	Dodecyl acrylate	C_15_H_28_O_2_	240.21	3.74	615.5	
	1,2-Benzenedicarboxylic acid, bis(2-methylpropyl) ester	C_16_H_22_O_4_	278.15	1.58	684.6	
	Pentadecanoic acid, 14-methyl-, methyl ester	C_17_H_34_O2	270.26	1.48	696.9	
	Diphenyl sulfone	C_12_H_10_O_2_S	218.04	59.23	725.1	
	1,6-Heptadiene, 2-methyl-6-phenyl-	C_14_H_18_	186.14	3.3	737.9	
	Pyridazin-3(2H)-one, 2-cyclohexyl-5-hydroxy-4-methoxy-	C_11_H_16_N_2_O_3_	224.12	1.48	763.7	
	Octadecanoic acid, methyl ester	C_19_H_38_O_2_	298.29	3.22	776.7	
	Hexadecanoic acid, 2-hydroxy-1-(hydroxymethyl)ethyl ester	C_19_H_38_O_4_	330.28	2.22	1022.6	
*D. bulbifera* bulb	1-(2-Aminoethylamino)-2-propanol	C_5_H_14_N_2_O	118.11	1.01	456.1	
	Ethyl acetate	C_4_H_8_O_2_	88.05	2.78	467.6	
	Ethyl acetate	C_4_H_8_O_2_	88.05	3.99	470.3	
	Diosgenin(3á,25R) acetate	C_2_9H_44_O_4_	456.32	57.29	699.9	
	Squalene	C_30_H_50_	410.39	18.86	760	

## Discussion

Free radicals are generated spontaneously as byproducts in biological systems during metabolic processes that can cause extensive damage to tissues and biomolecules leading to various severe clinical implications particularly diabetes mellitus, chronic inflammation, neurodegenerative disorders and cancer [29], [30]. Although a large number of synthetic drugs are proposed to protect against oxidative damage, a major drawback owing to adverse side effects restrict their use. Consumption of natural antioxidants from food supplements and traditional medicines constitute an alternative solution to the problem.

Phenolic and flavonoid compounds are reported to possess both potent antidiabetic and free radical scavenging activity [31]. Only preliminary report suggests that organic acid and polyphenols are present among *Dioscorea* spp. while there are no reports on *G. glauca* which might contribute to antioxidant properties. However, till date there are no reports on detailed study on different extracts of *Dioscorea bulbifera* and in-depth evaluation of different reactive oxygen species and free radical scavenging efficacy which can provide a strong rationale behind unexplored medicinal properties like antioxidative and antidiabetic potential [6], [21]. In our previous report we have established potent antidiabetic efficacy of *G. glauca* and *D. bulbifera* extracts [16]. Hereby, study of free radical scavenging activity is strongly rationalized and supported by report on antidiabetic and antioxidant potential of *Heliotropium zeylanicum* [32]. Total phenolic content estimation indicated higher concentration of polyphenols in methanolic and 70% (v/v) ethanolic extracts. Our results are concomitant with previous findings where high content of phenolics in alcoholic extract of *Moringa oleifera* leaves compared to aqueous extract was reported [33].

In addition, our findings on comparative analysis depict higher amount of phenolics in *G. glauca* leaf. Our results on free radical scavenging are in well agreement with amount of phenolic constituents present in respective extract. Phenolics present in *G. glauca* leaf extract are able to terminate radical chain reaction by converting free radicals to more stable products in greater extent, thus showing more activity as compared to other extracts. Free radical scavenging activity is characteristic to many traditional plants with antimicrobial as well as anticancer activity which is rationalized by reports on *Ficus asperifolia, Pentadesma butyracea, Psorospermum febrifugum, Rumex abyssinicus, Rumex bequaertii, Paullinia pinnata, Tectona grandis, Hibiscus asper, Dichrostachys glomerata* and *Origanum compactum* [34], [35]. It is important to note that *D. bulbifera* has been reported to possess both antimicrobial as well as anticancer activity [36], [37].

Similarly, high flavonoid content observed in ethyl acetate, methanolic and 70% (v/v) ethanolic extracts can contribute to enhanced antioxidant capacity as compared to petroleum ether extracts. Gao *et al,* has reported antitumor constituents in *D. bulbifera* which are flavonol glycosides, which can be a major contributory factor to antioxidant activity [15], [38]. Londhe *et al*, reported that polyphenol constituents of medicinal plant like *Phyllanthus amarus* Linn possess excellent property to scavenge pulse radiolysis generated ABTS ^+^ radical [21]. Similarly, flavonoids like quercetin 3-O-glucoside present in *Phyllanthus amarus* Linn was reported to show highest scavenging activity of pulse radiolysis generated hydroxyl radical [6]. Phenolic compounds are considered to be very important plant constituents responsible for free radical scavenging ability owing to their hydroxyl groups. In our present study it was observed that methanolic extract of *G. glauca* leaf showed maximum activity toward scavenging of DPPH, superoxide anion, superoxide radical, hydroxyl, nitric oxide radical. Similarly, it showed efficient ferric reducing antioxidant property and total reducing power. Our results are in well agreement with reports on *Drymaria diandra* Blume where methanolic extract of leaf showed higher activity owing to its high phenolic and flavonoid content [39]. Nitric oxide (NO) is a reactive free radical produced by phagocytes and endothelial cells, to yield more reactive species such as peroxynitrite which further decompose forming OH radical. *G. glauca* and *D. bulbifera* could significantly reduce the level of nitric oxide which is reported to play a crucial role in inflammation [40]. Thus, our findings strongly rationalize traditional use of both *G. glauca* and *D bulbifera* in inflammations and wound healing [11], [12], [41], [42].

Diphenyl sulfone was found to be predominant in extracts of *G. glauca.* There are very few reports on its natural occurrence, most noteworthy being in case of plant like *Myriactis humilis* [43]. It is significant to note that diphenyl sulfone and its derivatives has been reported as antioxidants and also used in treatment of various collagenoses of rheumatoid nature like rheumatoid arthritis as well as antioxidants [44], [45]. Hence, diphenyl sulfone can be probable phytochemical that might contribute to therapeutic potential of *G. glauca* in back ache and joint ache [12]. Diphenyl sulfone is a well know pesticide and hence its presence in *G. glauca* strongly rationalize mosquito larvicidal effects and ovicidal properties against eggs of teak defoliator, *Hyblaea puera* Cramer [11], [46]. Antibiotic nature of diphenyl sulfone similarly, supports antifungal activity of *G. glauca* [47].

Similarly, detection of diosgenin as major phytochemical in extracts of *D. bulbifera* highlights its significance as potent bioactive compound which is reported to be an antioxidant [48]. *In vivo* studies in both rat and mice models have proved that diosgenin rich extracts from *D. bulbifera* play the most significant role in antioxidant activity by upregulating antioxidant enzyme activities like total superoxide dismutase (SOD), peroxidase and catalase leading to cardioprotection by reducing lipid peroxidation, myocardial infarction as well as myocardial ischemic reperfusion injury [49], [50]. Other compounds detected in extracts like n-hexadecanoic acid, methyl ester of tridecanoic acid, 9,12,15-octadecatrienoic acid, (Z,Z,Z) and squalene were also reported for other medicinal plant like *Aloe vera L.* [28].

Thus our study provides strong scientific evidence for considering both *G. glauca* and *D. bulbifera* as natural antioxidants. Mechanism behind its significant medicinal importance is identified as scavenging of free radicals and thus preventing damage to biomolecules. Hence both the plants may be used for treatment in oxidative stress induced pathological conditions.

## Conclusion

*G. glauca* and *D. bulbifera* demonstrated rich phenolic and flavonoid content. A high positive correlation was observed between phytochemistry and free radical scavenging capacity. Our findings add one more attribute to spectrum of pharmacological properties supporting their use in traditional system of medicine. This is the first report on GC-TOF-MS identification of phytochemicals in *G. glauca* and *D. bulbifera*, which may rationalize and help in further *in-vivo* studies on oxidative stress and antioxidant activity with purified compounds.

## References

[1] Mohamed AA, Ali SI, El-Baz FK (2013) Antioxidant and antibacterial activities of crude extracts and essential oils of *Syzygium cumini* Leaves. PLoS One 8(4): e60269 10.1371/journal.pone.0060269 23593183 PMC3625181

[2] Sun J, Liu S-f, Zhang C-s, Yu L-n, Bi J, et al (2012) Chemical composition and antioxidant activities of *Broussonetia papyrifera* fruits. PLoS One 7(2): e32021 10.1371/journal.pone.0032021 22389678 PMC3289642

[3] Spanou C, Veskoukis AS, Kerasioti T, Kontou M, Angelis A, et al (2012) Flavonoid glycosides isolated from unique legume plant extracts as novel inhibitors of xanthine oxidase. PLoS One 7(3): e32214 10.1371/journal.pone.0032214 22396752 PMC3292553

[4] Box HC, Patrzyc HB, Budzinski EE, Dawidzik JB, Freund HG, et al (2012) Profiling oxidative DNA damage: Effects of antioxidants. Cancer Sci 103: 2002–2006. 22834775 10.1111/j.1349-7006.2012.02391.x PMC5439090

[5] Hazra B, Biswas S, Mandal N (2008) Antioxidant and free radical scavenging activity of *Spondias pinnata.* . BMC Complement Altern Med 8: 63. 19068130 10.1186/1472-6882-8-63 PMC2636748

[6] Londhe JS, Devasagayam TP, Foo LY, Ghaskadbi SS (2009) Radioprotective properties of polyphenols from *Phyllanthus amarus* Linn. J Radiat Res 50: 303–309. 19461166 10.1269/jrr.08096

[7] Shetty S, Udupa S, Udupa L (2008) Evaluation of antioxidant and wound healing effects of alcoholic and aqueous extract of *Ocimum sanctum* Linn in rats. Evid Based Complement Alternat Med 5: 95–101. 18317555 10.1093/ecam/nem004 PMC2249741

[8] Chang ST, Wu JH, Wang SY, Kang PL, Yang NS, et al (2001) Antioxidant activity of extracts from *Acacia confusa* bark and heartwood. J Agric Food Chem 49: 3420–3424. 11453785 10.1021/jf0100907

[9] Kähkönen MP, Hopia AI, Vuorela HJ, Rauha JP, Pihlaja K, et al (1999) Antioxidant activity of plant extracts containing phenolic compounds. J Agric Food Chem 47: 3954–3962. 10552749 10.1021/jf990146l

[10] Deepa VS, Kumar PS, Latha S, Selvamani S, Srinivasan S (2009) Antioxidant studies on the ethanolic extract of *Commiphora* spp. Afr J Biotechnol 8: 1630–1636.

[11] Amarajeewa BWRC, Mudalige AP, Kumar V (2007) Chemistry and mosquito larvicidal activity of *G. glauca* . In Proceedings of the Peradeniya University Research Sessions, SriLanka 12: 101–102.

[12] Kareru PG, Kenji GM, Gachanja AN, Keriko JM, Mungai G (2006) Traditional Medicines among the Embu and Mbeere peoples of Kenya. Afr J Tradit Complement Altern Med 4: 75–86. 20162075 10.4314/ajtcam.v4i1.31193 PMC2816425

[13] Kupchan SM, Shizuri Y, Sumner WC Jr, Haynes HR, Leighton AP, et al (1946) Isolation and structural elucidation of new potent antileukemic diterpenoid esters from *G.* Species. J Org Chem 41: 3850–3853. 10.1021/jo00886a016 993891

[14] Teklehaymanot T, Giday M (2007) Ethnobotanical study of medicinal plants used by people in Zegie Peninsula, Northwestern Ethiopia. J Ethnobiol Ethnomed 3: 12. 17355645 10.1186/1746-4269-3-12 PMC1852296

[15] Gao H, Kuroyanagi M, Wu L, Kawahara N, Yasuno T, et al (2002) Antitumor-promoting constituents from *D. bulbifera* L. in JB6. mouse epidermal cells. Biol Pharm Bull 25: 1241–1243. 12230129 10.1248/bpb.25.1241

[16] Ghosh S, Ahire M, Patil S, Jabgunde A, Bhat Dusane M, et al (2012) Antidiabetic activity of *Gnidia glauca* and *Dioscorea bulbifera*:Potent amylase and glucosidase inhibitors. Evid Based Complement Alternat Med 2012: 929051. 21785651 10.1155/2012/929051 PMC3140190

[17] Ghosh S, Patil S, Ahire M, Kitture R, Gurav DD, et al (2012) *Gnidia glauca* flower extract mediated synthesis of gold nanoparticles and evaluation of its chemocatalytic potential. J Nanobiotechnology 10: 17. 22548753 10.1186/1477-3155-10-17 PMC3462129

[18] Ghosh S, Patil S, Ahire M, Kitture R, Kale S, et al (2012) Synthesis of silver nanoparticles using *Dioscorea bulbifera* tuber extract and evaluation of its synergistic potential in combination with antimicrobial agents. Int J Nanomedicine 7: 483–496. 22334779 10.2147/IJN.S24793 PMC3273981

[19] Ghosh S, Patil S, Ahire M, Kitture R, Jabgunde A, et al. (2011) Green synthesis of gold nano-anisotrops using *Dioscorea bulbifera* tuber extract. J Nanomater 2011:doi:10.1155/2011/354793.

[20] Luximon-Ramma A, Bahorun T, Soobrattee MA, Aruoma O (2002) Antioxidant activities of phenolic, proanthocyanidin and flavonoid components in extracts of *Cassia fistula* . J Agric Food Chem 50: 5042–5047. 12188605 10.1021/jf0201172

[21] Londhe JS, Thomas PA, Devasagayam TP, Foo LY, Ghaskadbi SS (2008) Antioxidant activity of some polyphenol constituents of the medicinal plant *Phyllanthus amarus* Linn. Redox Rep 13: 199–207. 18796238 10.1179/135100008X308984

[22] Jiao Z, Liu J, Wang S (2005) Antioxidant activities of total pigment extract from blackberries. Food Technol Biotech 43: 97–102.

[23] Rahman MAA, Moon SS (2007) Antioxidant polyphenol glycosides from the plant *Draba nemorosa.* . Bull *Korean Chem Soc* 28: 827–831.

[24] Pulido R, Bravo L, Saura-Calixto F (2000) Antioxidant activity of dietary polyphenols as determined by a modified ferric reducing/antioxidant power assay. J Agric Food Chem 48: 3396–3402. 10956123 10.1021/jf9913458

[25] Tan M, Liu Y, Luo X, Chen Z, Liang H (2011) Antioxidant activities of plumbagin and its Cu (II) complex. Bioinorg Chem Appl 2011: 898726 10.1155/2011/898726 22046145 PMC3199187

[26] Kitture R, Ghosh S, Kulkarni P, Liu XL, Maity D, et al (2012) Fe_3_O_4_-citrate-curcumin: Promising conjugates for superoxide scavenging, tumor suppression and cancer hyperthermia. J Appl Phys 111: 064702–064707.

[27] Marcocci L, Packer L, Droy-Lefaix MT, Sekaki A, Gardes-Albert M (1994) Antioxidant action of *Ginkgo biloba* extract EGB 761. Methods Enzymol 234: 462–475. 7808320 10.1016/0076-6879(94)34117-6

[28] Arunkumar S, Muthuselvam M (2009) Analysis of phytochemical constituents and antimicrobial activities of *Aloe vera* L. against clinical pathogens. World J Agric Sci 5: 572–576.

[29] Frankel N, German JB (2006) Antioxidants in foods and health: problems and fallacies in the field. J Sci Food Agric 86: 1999–2001.

[30] Yazdanparast R, Bahramikia S, Ardestani A (2008) *Nasturtium oficinale* reduces oxidative stress and enhances antioxidant capacity in hypercholesterolaemic rats. Chem Biol Interact 172: 176–184. 18325487 10.1016/j.cbi.2008.01.006

[31] Samane S, Noël J, Charrouf Z, Amarouch H, Haddad PS (2006) Insulin-sensitizing and anti-proliferative effects of *Argania spinosa* seed extracts. Evid Based Complement Alternat Med 3: 317–327. 16951716 10.1093/ecam/nel015 PMC1513146

[32] Murugesh K, Yeligar V, Dash DK, Sengupta P, Maiti BC, et al (2006) Antidiabetic, antioxidant and antihyperlipidemic status of *Heliotropium zeylanicum* extract on streptozotocin-induced diabetes in rats. Biol Pharm Bull 29: 2202–2205. 17077515 10.1248/bpb.29.2202

[33] Siddhuraju P, Becker K (2003) Antioxidant properties of various solvent extracts of total phenolic constituents from three different agroclimatic origins of drumstick tree (*Moringa oleifera* Lam.) leaves. J Agric Food Chem 51: 2144–2155. 12670148 10.1021/jf020444+

[34] Tamokou JdD, Chouna JR, Fischer-Fodor E, Chereches G, Barbos O, et al (2013) Anticancer and antimicrobial activities of some antioxidant-rich Cameroonian medicinal plants. PLoS One 8(2): e55880 10.1371/journal.pone.0055880 23409075 PMC3569468

[35] Bouhdid S, Skali SN, Idaomar M, Zhiri A, Baudoux D, et al (2008) Antibacterial and antioxidant activities of *Origanum compactum* essential oil. Afr J Biotechnol 7: 1563–1570.

[36] Teponno RB, Tapondjou AL, Gatsing D, Djoukeng JD, Abou-Mansour E, et al (2006) Bafoudiosbulbins A, and B, two anti salmonellal clerodane diterpenoids from *Dioscorea bulbifera* L. var sativa. Phytochemistry 67: 1957–1963. 16876211 10.1016/j.phytochem.2006.06.019

[37] Liu H, Chou GX, Wu T, Guo YL, Wang SC, et al (2009) Steroidal sapogenins and glycosides from the rhizomes of *Dioscorea bulbifera* . J Nat Prod 72: 1964–1968. 19842682 10.1021/np900255h

[38] Gao H, Hou B, Kuroyanagi M, Wu L (2007) Constituents from anti-tumor-promoting active part of *Dioscorea bulbifera* L. in JB6 mouse epidermal cells. Asian J Tradit Med 2: 104–109.

[39] Mandal P, Misra TK, Ghosal M (2009) Free-radical scavenging activity and phytochemical analysis in the leaf and stem of *Drymaria diandra* Blume. Int J Integr Biol 7: 80–84.

[40] Moncada S, Palmer RM, Higgs EA (1991) Nitric oxide: Physiology, pathophysiology and pharmacology. Pharmacol Rev 43: 109–142. 1852778

[41] Mbiantcha M, Kamanyi A, Teponno RB, Tapondjou AL, Watcho P, et al. (2011) Analgesic and anti-Inflammatory properties of extracts from the bulbils of *D. bulbifera* L. var sativa [D.ceae] in mice and rats. Evid Based Complement Alternat Med 2011:doi:10.1155/2011/912935. PMC2952333 20953397

[42] Nguelefack TB, Dutra RC, Paszcuk AF, Andrade EL, Tapondjou LA, et al (2010) Antinociceptive activities of the methanol extract of the bulbs of *Dioscorea bulbifera* L. var sativa in mice is dependent of NO–cGMP–ATP-sensitive-K+ channel activation. J Ethnopharmacol 128: 567–574. 20152893 10.1016/j.jep.2010.01.061

[43] Chen JJ, Duh CY, Chen IS (2005) Cytotoxic chromenes from *Myriactis humilis* . Planta Med 71: 370–372. 15856418 10.1055/s-2005-864107

[44] Diaz-Ruiz A, Zavala C, Montes S, Ortiz-Plata A, Salgado-Ceballos H, et al (2008) Antioxidant, antiinflammatory and antiapoptotic effects of Dapsone in a model of brain ischemia/reperfusion in rats. Journal of Neurosci Res 86: 3410–3419. 18615706 10.1002/jnr.21775

[45] Goloschapov NM, Sigidin YA, Tsvetkova ES, Bilich IL., Reznik VS, et al.. (1979) Medicinal preparation for the treatment of collagenoses of a rheumatoid nature. U.S. Patent number: 4151281, 1979.

[46] Javaregowda K, Naik LK (2007) Ovicidal properties of plant extracts against the eggs of teak defoliator, *Hyblaea puera* Cramer. Karnataka J Agric Sci 20: 291–293.

[47] Naik ST, Maheswarappa V (2007) Prospects of using plant extracts in management of pineapple heart rot. Karnataka J Agric Sci 20: 180–182.

[48] Patel K, Gadewar M, Tahilyani V, Patel DK (2012) A review on pharmacological and analytical aspects of diosgenin: a concise report. Nat Products Bioprospect 2: 46–52.

[49] Balasubramanian J, Dhanalakshmi R, Jibnomen J, Manimekalai P (2012) A preclinical evaluation on antioxidant and gastroprotective effect of *Dioscorea bulbifera* in Wistar rats. Indian J Innovations Dev 1: 149–154.

[50] Vasanthi HR, ShriShriMal N, Das DK (2012) Phytochemicals from plants to combat cardiovascular disease. Curr Med Chem 19: 2242–2251. 22414106 10.2174/092986712800229078

